# The correlation between intracranial atherosclerosis and white matter hyperintensities in cerebral small vessel disease: a high-resolution magnetic resonance imaging study

**DOI:** 10.3389/fneur.2024.1485921

**Published:** 2025-01-10

**Authors:** Gui-Song Zhang, Wei Bu, Ling-Hui Meng, Wen-Jun Li, Yu-Juan Dong, Xiao-Yun Cao, Qi Gao, Xuan-Ye Zhang, Hui-Ling Ren

**Affiliations:** ^1^Department of Neurology, The Third Hospital of Hebei Medical University, Shijiazhuang, China; ^2^Department of Neurosurgery, The Third Hospital of Hebei Medical University, Shijiazhuang, China; ^3^Department of Radiology, The Third Hospital of Hebei Medical University, Shijiazhuang, China; ^4^School of Basic Medicine, Hebei Medical University, Shijiazhuang, China

**Keywords:** white matter hyperintensities, middle cerebral artery, atherosclerosis, high-resolution magnetic resonance imaging, volume, ischemic stroke, positive remodeling

## Abstract

**Objective:**

Recent studies have indicated a close relationship between intracranial arterial stenosis and white matter hyperintensities (WMHs), but few have reported on the correlation between the characteristics of intracranial arterial wall plaques and WMHs. The aim of this study was to comprehensively assess the correlation between intracranial atherosclerosis plaques and WMHs using 3.0T high-resolution magnetic resonance imaging (HR-MRI).

**Patients and methods:**

Ninety-two ischemic stroke patients with middle cerebral artery (MCA) stenosis <50% on cranial magnetic resonance angiography (MRA) underwent conventional MRI and HR-MRI examinations. T2-weighted fluid-attenuated inversion recovery (T2-FLAIR) images were processed using 2D VBNeT automatic segmentation technology to segment WMH volume. HR-MRI images were analyzed using ImageJ software to evaluate the luminal area, outer wall area, plaque distribution, luminal stenosis rate, remodeling patterns, and other plaque parameters at the stenosis site and reference points of the MCA M1 segment. The correlation between the presence of plaques, plaque distribution, luminal stenosis rate, T1 hyperintensity, remodeling patterns, remodeling ratio (RR), eccentric plaques, and plaque burden with the volume of cerebral WMHs was analyzed.

**Results:**

Compared with the no-plaque group, the plaque group had significantly higher age, male ratio, total WMH volume, periventricular WMH (PVWMH) volume, deep WMH (DWMH) volume, and juxtacortical WMH (JCWMH) volume (all *p* < 0.05). The Kruskal-Wallis H test showed that patients with plaques on the superior and dorsal sides of the MCA M1 segment had higher levels of total WMH volume, PVWMH volume, DWMH volume, and JCWMH volume than patients with plaques on the inferior and ventral sides (all *p* < 0.05). Age, diabetes, previous stroke events, plaque distribution, positive remodeling, eccentric plaques, and RR were positively correlated with total WMH volume, PVWMH volume, and JCWMH volume (all *p* < 0.05). The presence of plaques, plaque distribution, and positive remodeling were independent risk factors for total WMH volume (all *p* < 0.05).

**Conclusion:**

The presence of intracranial atherosclerotic plaques, plaque distribution, and positive remodeling are closely associated with increased cerebral WMHs burden in patients with ischemic stroke, which further supports the relationship between large artery atherosclerosis and CSVD.

## Highlights

- Question: Intracranial atherosclerosis and white matter hyperintensities (WMHs) are commonly found in patients with ischemic stroke. However, the relationship between the characteristics of intracranial arterial wall plaques and WMHs remains unclear.
- Findings: In patients with ischemic stroke, the characteristics of intracranial arterial wall plaques, such as plaque distribution and positive remodeling, are closely associated with the development of WMHs.
- Meaning: Research based on HR-MRI indicates that the characteristics of intracranial atherosclerotic plaques in patients with ischemic stroke can exacerbate the white matter burden, providing a theoretical foundation for the effective prevention and treatment of WMHs.

## 1 Introduction

Cerebral small vessel disease (CSVD) is characterized by affecting the perforating cerebral arterioles, capillaries, and venules, which can lead to a variety of clinical manifestations and imaging features. White matter hyperintensities (WMHs) serve as a critical imaging biomarker that reflects the underlying vascular pathology of CSVD, while the etiology and mechanisms of WMHs are multifactorial. In the context of CSVD, ischemia triggers a series of pathophysiological alterations, the breakdown of the blood–brain barrier, facilitating the infiltration of inflammatory mediators and exacerbating the tissue damage (1), thereby impairing the normal functioning of the brain’s white matter tracts.

Numerous studies have confirmed the coexistence of atherosclerotic stenosis with cerebral WMHs (2). A large artery and small vessel disease may share common vascular risk factors, such as advanced age, hypertension, and diabetes, that could play a mediating role. A meta-analysis showed that the presence of ≥50% atherosclerotic stenosis in both intracranial and extracranial arteries was associated with an increased risk of WMHs and increased volume of WMHs, even after accounting for the effects of vascular risk factors (3). Another CT-based brain perfusion study found WMHs attributable to cerebral hypoperfusion secondary to cranial vascular stenosis (4). Thus, the pathophysiological link between the two is thought to be a reduction in cerebral blood flow, leading to chronic ischemia in the white matter.

In addition to atherosclerotic stenosis of the large cerebral arteries, plaque composition characteristics have been reported to be strongly associated with the severity of WMHs (5). A CARE-II study (6) showed that carotid atherosclerotic plaque characteristics were more useful in assessing the severity of WMHs than the degree of stenosis within intracranial arteries. In recent years, the presence of atherosclerotic plaques (especially complex plaques) has also been shown to be strongly associated with imaging markers of CSVD (7). High-resolution magnetic resonance imaging (HR-MRI) allows non-invasive visualization of intracranial vessel walls, making it possible to comprehensively explore the structure and composition of unstable plaques. An HR-MRI study discussed the significant role of intracranial arterial vulnerable plaque characteristics, particularly intraplaque hemorrhage, and plaque enhancement in the severity of WMHs (8). Therefore, the study provides us with a new perspective to explore the pathophysiological mechanisms of CSVD. In addition to alterations in cerebral hemodynamics, mechanisms such as inflammatory responses, microembolism, and vascular dysfunction are likely to contribute to disease progression.

Therefore, based on the HR-MRI study, we deeply assess the presence and characteristics of atherosclerotic plaques in the middle cerebral artery (MCA) and their association with WMHs. The aim was to identify potential independent risk factors for the volume of WMHs, and further explore the underlying vascular pathology of CSVD.

## 2 Methods

### 2.1 Patients

Patients were selected from the Third Hospital of Hebei Medical University. A retrospective study of consecutive patients with cerebral ischemic stroke who underwent HR-MRI was performed between January 2021 and April 2024. Inclusion criteria were: (1) age ≥ 18; (2) diffusion-weighted imaging (DWI) showed evidence of acute infarct foci in the blood-supplying region of the MCA; (3) MRI was performed within 2 weeks of symptoms onset; (4) presence of at least one atherosclerosis risk factor; (5) all the enrolled patients underwent HR-MRI, T2-weighted fluid-attenuated inversion recovery (T2-FLAIR) and magnetic resonance angiography (MRA). Exclusion criteria were: (1) ≥ 50% stenosis of one carotid artery or MCA on brain MRA; (2) presence of nonatherosclerotic vascular diseases, such as vasculitis, moyamoya, dissection, arterial sequestration, etc.; (3) presence of massive cerebral infarction, intracerebral hemorrhage, intracranial infections, intracranial tumor, multiple sclerosis, cerebral white matter dystrophy, other CNS demyelinating diseases and degenerative diseases, etc.; (4) presence of contraindications to MRI; (5) poor image quality of HR-MRI or conventional MRI for accurate quantitative analysis of dilated arterial borders.

The basic clinical information was collected, including age, sex, hypertension, diabetes, coronary artery disease, hyperlipidemia, previous stroke events, smoking, drinking, and systolic and diastolic blood pressure on admission. The next morning after admission, fasting venous blood was collected from all the enrolled patients and then sent to the clinical laboratory, the levels for fasting plasma total cholesterol (TC), triglyceride (TG), high-density lipoprotein (HDL), low-density lipoprotein (LDL), very low-density lipoprotein (VLDL), homocysteine (HCY) and uric acid (UA) were recorded.

### 2.2 MR imaging protocol

All participants underwent magnetic resonance imaging at the Third Hospital of Hebei Medical University with a 3.0 T MRI scanner (Signal Architect, GE Medical Systems, America) with a 19-channel head–neck coil. Conventional MRI and HR-MRI scanning protocols are included: T2-FLAIR, DWI, three-dimensional time-of-flight MRA (3D-TOF MRA), 3D CUBE T1-weighted imaging (3D CUBE T1WI), and 3D proton density-weighted imaging CUBE (3D PDWI CUBE). Sequence parameters for T2-FLAIR imaging were as follows: Repetition time (TR) = 9,000 ms; echo time (TE) = 100 ms; slice thickness = 5 mm; slices = 23; field of view (FOV) = 240 × 240 mm2; matrix = 288 × 200; pixel = 0.8 × 1.2. Sequence parameters for DWI imaging were as follows: TR = 4,351 ms; TE = 76 ms; slice thickness = 5 mm; slices = 23; FOV = 240 × 240 mm2; matrix = 128 × 160; pixel = 1.9 × 1.5. Sequence parameters for 3D-TOF MRA imaging were as follows: TR = 19 ms; TE = 3.4 ms; slice thickness = 0.7 mm; slices = 168; FOV = 220 × 220 mm2; matrix = 384× 160; pixel = 0.6 × 1.4; voxel size = 0.6 × 1.4 × 1.4. Sequence parameters for 3D CUBE T1WI imaging were as follows: TR = 702 ms, TE = 13.27 ms, slice thickness = 0.4 mm; slices = 272; FOV = 200 × 200 mm2; matrix = 280 × 280; pixel = 0.7 × 0.7; voxel size = 0.7 × 0.7 × 0.8. Sequence parameters for 3D PDWI CUBE imaging were as follows: TR = 1,500 ms, TE = 40 ms, slice thickness = 0.4 mm; slices = 272; FOV = 200 × 200 mm2; matrix = 260 × 260; pixel = 0.8 × 0.8; voxel size = 0.8 × 0.8 × 0.8. The use of a 3D-TOF MRA localizer ensured that HR-MRI cross-sectional images were perpendicular to the M1 segment of the middle cerebral artery.

### 2.3 MR image analysis

All patient MRI images were reviewed by two experienced neuroradiologists (M.LH and R.BB, each with 5 years of experience in neuroimaging), both of them were unaware of demographic and clinical information, and peer-reviewed by another senior neuroradiologist (P.ZG, 10 years of experience in neuroimaging) in cases of disagreement in the image analysis results. Evaluators used ImageJ software designed by the National Institutes of Health for post-processing.

### 2.4 Assessment of plaque characteristics

The image quality of the intracranial arterial vessel wall images was assessed and the image quality was categorized into four classes (9): Grade 1, the borders of the vessel lumen and outer wall were poorly displayed, severe artifacts were present, and the signal characteristics of the wall could not be analyzed; Grade 2, some of the borders of the vessel lumen and outer wall were displayed, a few artifacts were present, and the signal characteristics of the wall still could not be analyzed; Grade 3, the borders of the vessel lumen and outer wall were displayed, a few artifacts were present, and the signal characteristics of the wall could be analyzed; Grade 4, the borders of the vessel lumen and outer wall were displayed, no artifacts were present, and the signal characteristics of the vessel wall could be analyzed. MRI images with a grade of ≥2 were included.

**Figure 1:**
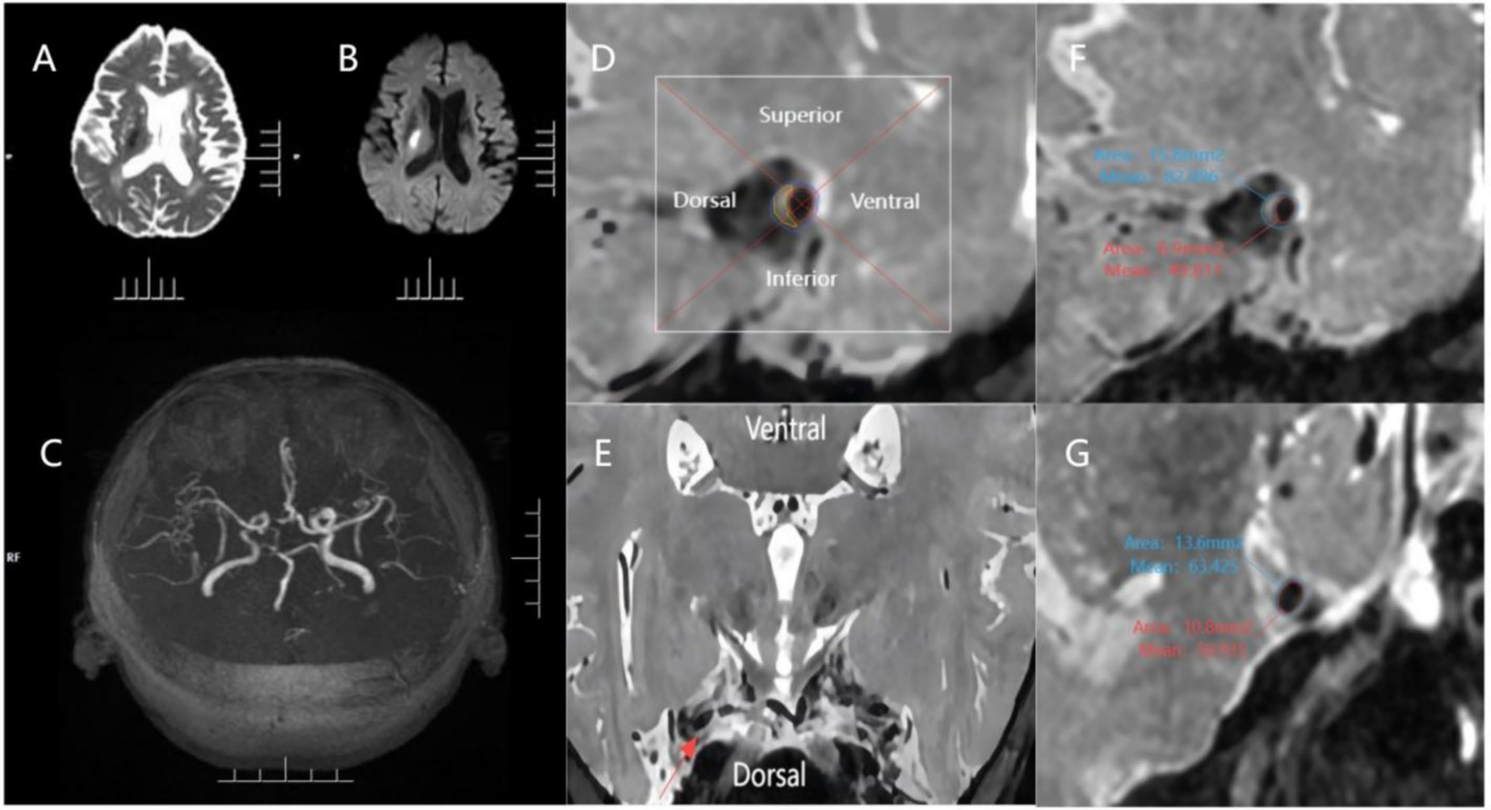
An example of evaluating plaque characteristics. The patient is a 70-year-old male who has experienced an acute ischemic stroke. **(A,B)** Diffuse right basal ganglia area is confined, with the hypointense signal on the ADC map and confined hyperintense signal on the b = 1,000 map; **(C)** 3D-TOF-MRA image showing mild stenosis of the M1 segment of middle cerebral artery bilaterally; **(D,E)** The cross-section of the plaque was divided into 4 quadrants (superior, inferior, ventral, and dorsal) using 2 perpendiculars dashed lines intersecting at the center of the lumen, as shown in the magnified image of the plaque. The red line indicates the lumen area, the blue line indicates the outer wall area and the yellow line indicates the estimated plaque. The relevant quadrant of the plaque was further verified using the reconstructed axial view of the MCA wall; **(F)** 3D PDWI CUBE measurements of the narrowest portion of the MCA M1 segment plaque: OWA 15.80 mm 2, LA 6.90 mm 2; **(G)** 3D PDWI CUBE measurement of the reference site: OWA 13.60 mm 2, LA 10.80. Remodeling ratio = 15.80 mm 2 / 13.60 mm 2 = 1.16 (positive remodeling); degree of stenosis = (1–6.90 mm 2 / 10.80 mm 2) = 36.11%.

Plaque assessment of MCA was performed using 3D CUBE T1WI and 3D PDWI CUBE sequences. Using multiplanar reconstruction (MPR), the three-dimensional localization points were adjusted to be located in the center of the lumen in sagittal, coronal, and axial positions. And at the same time, the cross-sections were adjusted to be perpendicular to the long axis of the vessel, to obtain a cross-sectional image of the stenosis at the narrowest point of the M1 segment of MCA. Plaque characteristics were analyzed based on cross-sectional images: (1) To determine the presence or absence of plaque in the MCA M1 segment of the subjects; (2) To assess the distribution of plaque in the narrowest part of the MCA M1 segment: on the cross-sectional images, centered on the lumen, it was divided into 4 quadrants equally: including the superior wall, the inferior wall, the ventral wall, and the dorsal wall. Quadrants accrued by plaques were recorded, and when 2 or more quadrants were accrued, the quadrant with the greatest plaque thickness was selected (10); (3) Determination of the presence of intraplaque hemorrhage in MCA M1 segment plaques: intraplaque hemorrhage was considered to be present when there was a high-signal area within the plaque at the narrowest point of the MCA M1 segment on the 3D CUBE T1WI image (with a signal intensity of >150% of 150% of the area of the adjacent vessel wall of the reference wall) (11); (4) Analysis of quantitative plaque characteristics: the vessel with the largest stenosis in the MCA M1 segment was selected, the reference vessel was preferred to be a no-plaque normal vessel proximal to the level of the largest stenosis, and if the proximal reference vessel was unavailable, an adjacent distal no-plaque normal vessel was used. Cross-sectional images of the lumen were magnified by 300% by two neuroradiologists. The lumen and wall borders were manually outlined on ImageJ software according to the region of interest (ROI), the outer wall area (OWA), the lumen area (LA), the maximum wall thickness, and the minimum wall thickness of the largest stenosis in the MCA M1 segment and the reference vessel were measured and recorded semi-automatically, respectively. The degree of lumen stenosis, remodeling ratio, eccentricity, and plaque burden were calculated based on the measurement results. The formulas for the relevant parameters were as follows: degree of luminal stenosis = (1 - LA stenosis / LA reference) × 100% (MCA stenosis was classified as mild stenosis 0–49%, moderate stenosis 50–69%, and severe stenosis 70–99%) (12); remodeling ratio (RR) = OWA stenosis / OWA reference (RR > 1.05 indicates positive remodeling; 0.95 ≤ RR ≤ 1.05 indicates no remodeling; RR < 0.95 indicates negative remodeling) (13); eccentric plaques (eccentricity ≥0.5) (eccentricity = (maximum wall thickness - minimum wall thickness) / maximum wall thickness (14)); plaque burden was quantified using the normal wall index (NWI), plaque burden = (OWA stenosis - LA stenosis) / OWA stenosis (15) (Figure 1).

### 2.5 WMH volume measurements

**Figure 2:**
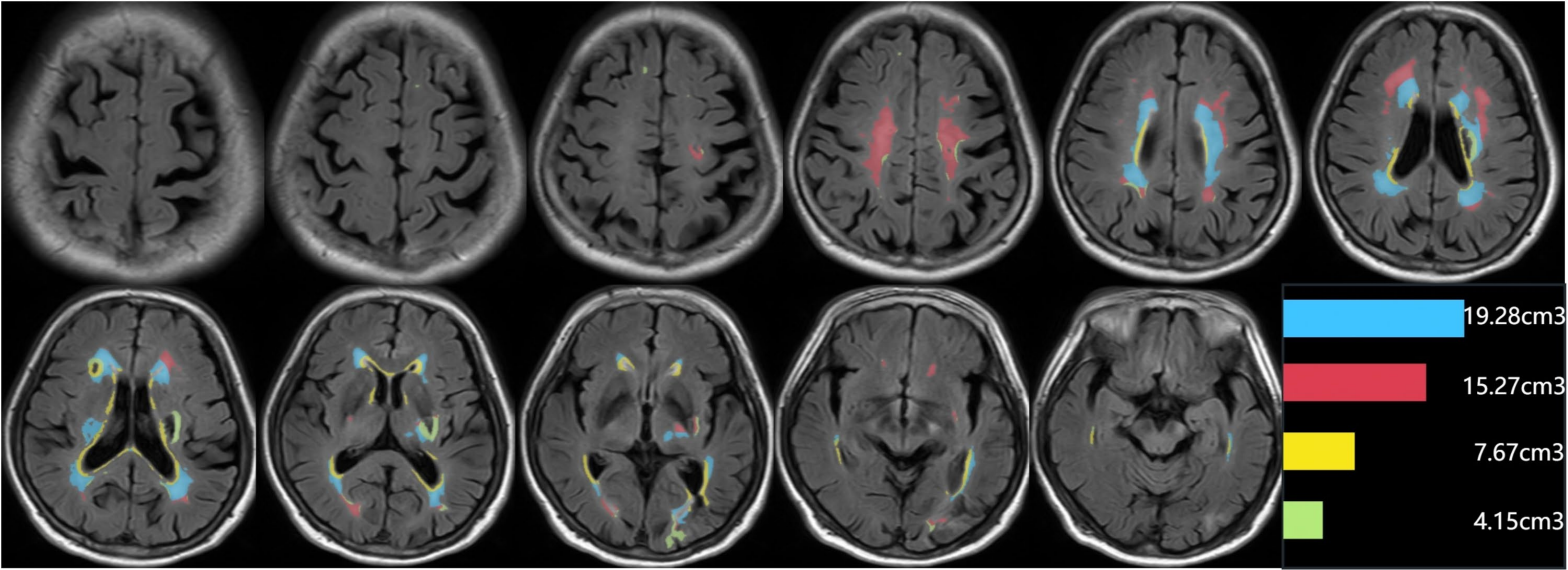
An example of segmentation results for subclassified WMHs. Display the segmentation map and the volumes of each subclassified WMH area. The regions of each subclassified WMH are represented by different colors (blue zone = lateral paraventricular WMH volume; red zone = deep WMH volume; yellow zone = lateral ventricular rim WMH volume; green = juxtacortical WMH volume).

The T2-FLAIR images were collected and downloaded in Dic.com format before uploading them to the United Imaging Intelligence MR CSVD Intelligent Analysis System for post-processing (16) which uses a convolutional neural network algorithm with a 2D VB-Net to segment the WMHs, and which outperforms the other state-of-the-art methods in segmenting the WMHs (such as HughesNet, 3D V-Net, and VisualGeometryGroup Network). The total WMH volume (total WMH volume = periventricular WMH volume + deep WMH volume + juxtacortical WMH volume), periventricular WMH volume (PVWMH, PVWMH = lateral ventricular rim WMH volume + lateral paraventricular WMH volume), deep WMH volume (DWMH), and juxtacortical WMH volume (JCWMH) were calculated (Figure 2).

### 2.6 Statistical analysis

We used a statistical package for the social sciences (SPSS 26) to conduct statistical analysis and used GraphPad Prism 9 to plot. When comparing the baseline characteristics and imaging characteristics of the plaque group and the no-plaque group. Continuous variables that conformed to normal distribution were expressed as mean ± standard deviation. Continuous variables that were not normally distributed were expressed as median and interquartile spacing, and categorical variables were expressed as frequency and percentage. Continuous variables were tested using the student-*t* test or the Mann–Whitney *U* test, and categorical variables were tested using the *χ*2 test or the Fisher exact test. For the plaque group, the Kruskal-Wallis H test was used to compare the relationship between the quadrant in which the different plaques were located and the WMH volume. Spearman correlation analysis was used to determine the relationship of clinical characteristics and plaque characteristics with total WMH volume, PVWMH volume, DWMH volume, and JCWMH volume. Independent variables controlling for *p* < 0.1 in Spearman’s correlation analysis were included in a multifactorial generalized linear model to analyze the relationship between different plaque parameters and WMH volume. The variance inflation factors (VIFs) among variables were checked to avoid multicollinearity among independent variables. Two-tailed *p*-values less than 0.05 were considered statistically significant.

## 3 Results

### 3.1 Clinical characteristics

**Figure 3:**
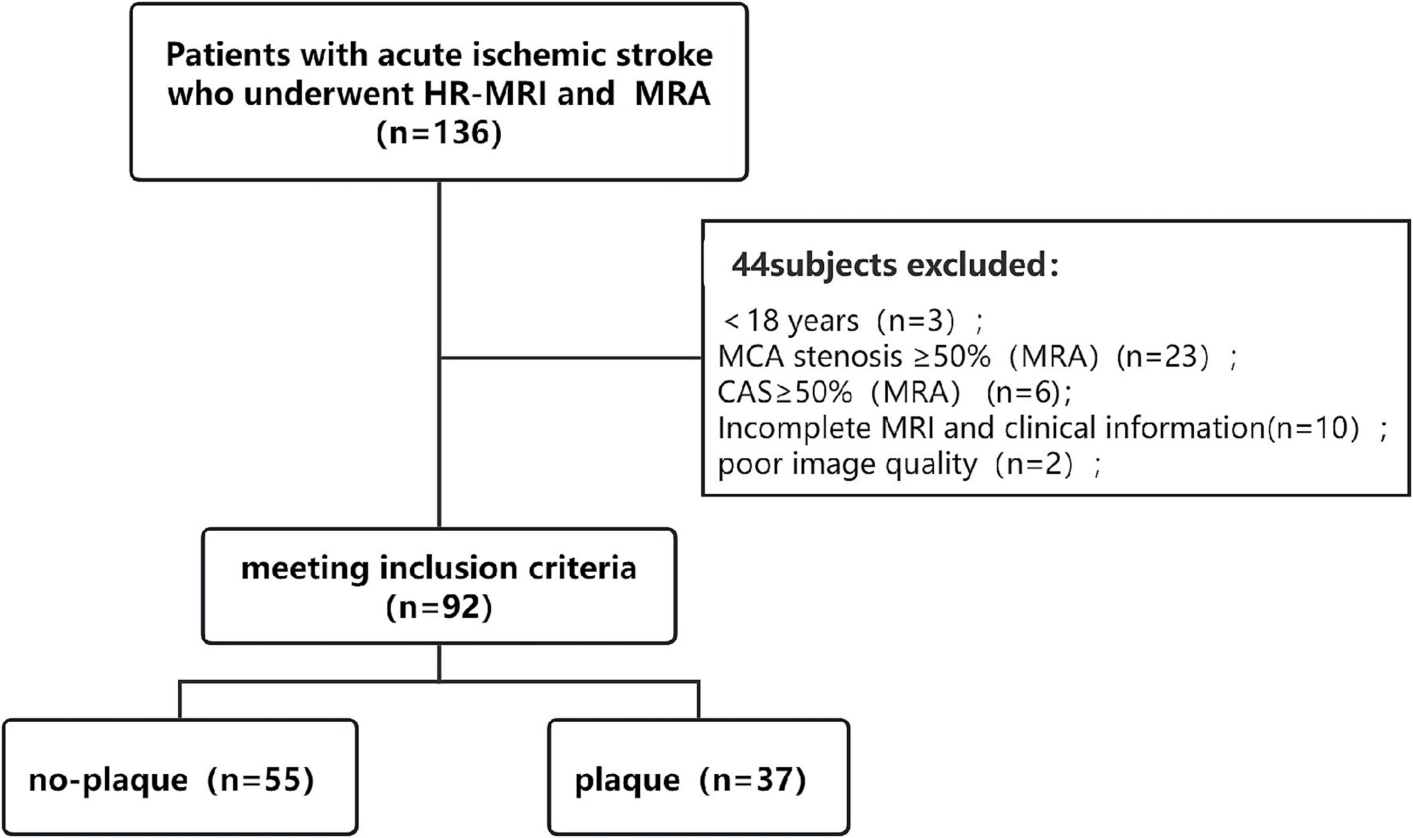
Flow chart. HR-MRI, high-resolution magnetic resonance imaging; MRA, magnetic resonance angiography; MCA, middle cerebral artery; CAS, carotid artery stenosis.

A total of 136 patients with acute ischemic stroke who visited our hospital from January 2021 to May 2024 were included. Among them, 92 patients met the inclusion and exclusion criteria for enrollment. The flow chart of subject enrolment is shown (Figure 3). The 92 patients finally included in the statistical analysis had a mean age of 55.50 ± 12.87 years, 64 males, 65 with hypertension, 29 with diabetes, 12 with coronary artery disease, 21 with hyperlipidemia, 19 smokers, 21 drinkers, and 32 previous strokes. Among the subjects of this study, no-plaque was seen in the MCA M1 segment in 55 patients (59.78%) and the plaque was present in the MCA M1 segment in 37 patients (40.22%).

### 3.2 Comparison of clinical and imaging characteristics between plaque group and no-plaque group

**Figure 4:**
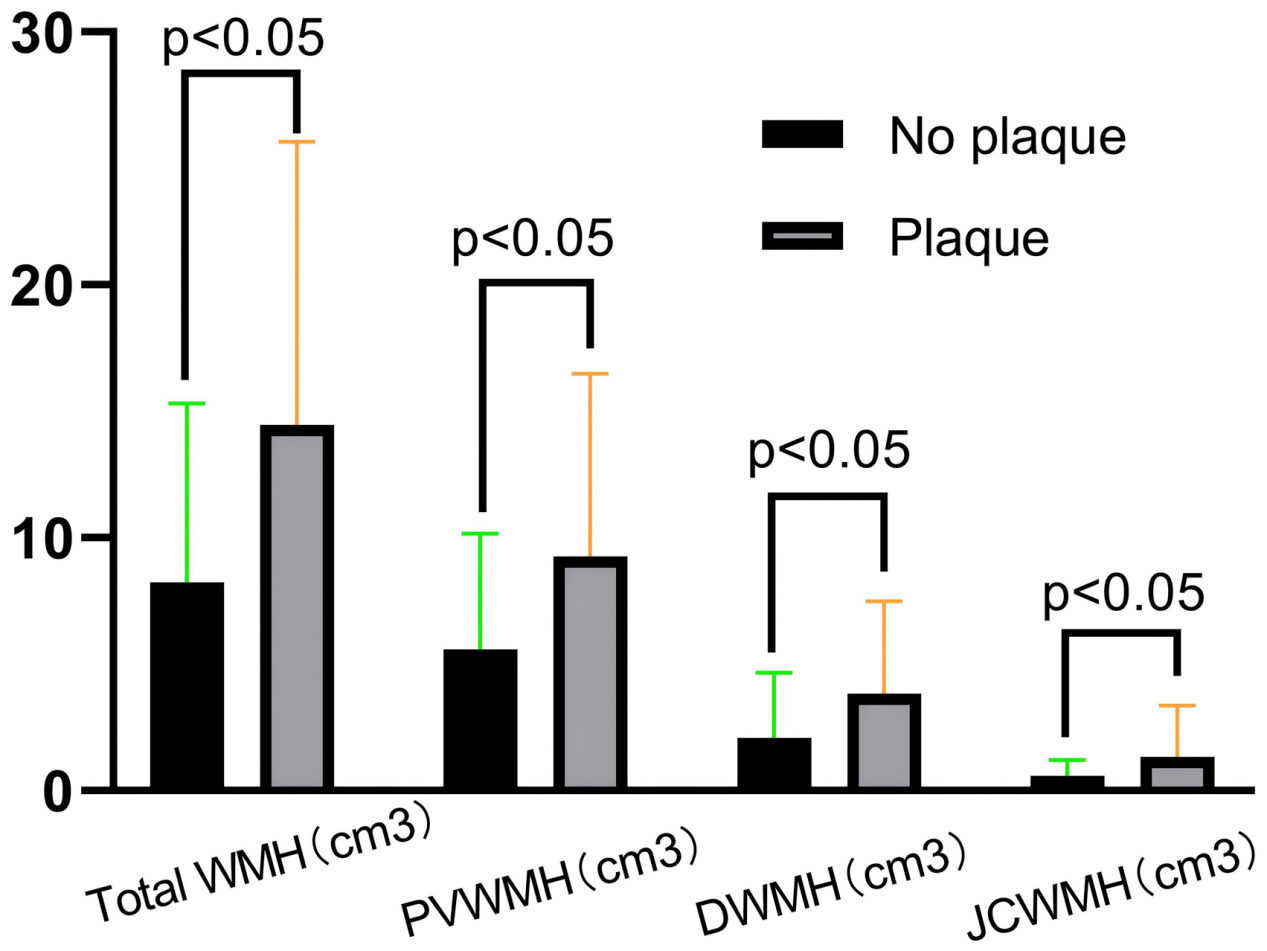
Imaging characteristics of patients between the plaque group and the no-plaque group.

Compared to the no-plaque group (*n* = 55), the plaque group (*n* = 37) had higher levels of age, percentage of males, total WMH volume, PVWMH volume, DWMH volume, and JCWMH volume (Table 1 and Figure 4) (all *p* < 0.05).

**Table 1:** Comparison of clinical and imaging characteristics between the plaque group and the no-plaque group.

Characteristic	No-plaque group (*n* = 55)	Plaque group (*n* = 37)	*p*-value
Age (years), mean [SD]	53.33 ± 13.09	58.73 ± 12.00	0.048
Sex male, *n* (%)	34 (61.8%)	30 (81.1%)	0.049
Vascular risk factor			
Hypertension, *n* (%)	37 (67.3%)	28 (75.7%)	0.385
Diabetes, *n* (%)	14 (25.5%)	15 (40.5%)	0.127
Coronary artery disease, *n* (%)	5 (9.1%)	7 (18.9%)	0.170
Hyperlipidemia, *n* (%)	11 (20.0%)	10 (27.0%)	0.431
Smoking, *n* (%)	8 (14.6%)	11 (29.7%)	0.078
Drinking, *n* (%)	9 (16.4%)	12 (32.4%)	0.072
Previous stroke events, *n* (%)	18 (32.7%)	14 (37.8%)	0.614
Laboratory parameters			
Systolic blood pressure, mean [SD], mmHg	138.49 ± 20.41	144.92 ± 21.15	0.148
Diastolic blood pressure, mean [SD], mmHg	87.35 ± 18.03	88.68 ± 12.81	0.700
HCRP, median [IQR], mmol/L	2.49 (1.11, 8.24)	2.07 (1.11, 6.13)	0.516
Hcy, median [IQR], mmol/L	12.30 (10.50, 17.19)	13.20 (10.79, 16.55)	0.946
TC, median [IQR], mmol/L	4.56 (3.66, 5.32)	4.56 (3.96, 5.22)	0.933
TG, median [IQR], mmol/L	1.35 (1.04, 1.84)	1.33 (0.98, 2.29)	0.729
HDL, median [IQR], mmol/L	1.07 (0.90, 1.28)	1.00 (0.89, 1.18)	0.534
LDL, mean [SD], mmol/L	2.66 ± 0.81	2.58 ± 0.72	0.602
VLDL, median [IQR], mmol/L	0.61 (0.47, 0.84)	0.60 (0.45, 1.04)	0.723
UA, median [IQR], mmol/L	328.00 (276.00, 397.00)	337.00 (261.50, 414.50)	0.927

### 3.3 Comparison of WMH volume for different plaque distributions

**Figure 5:**
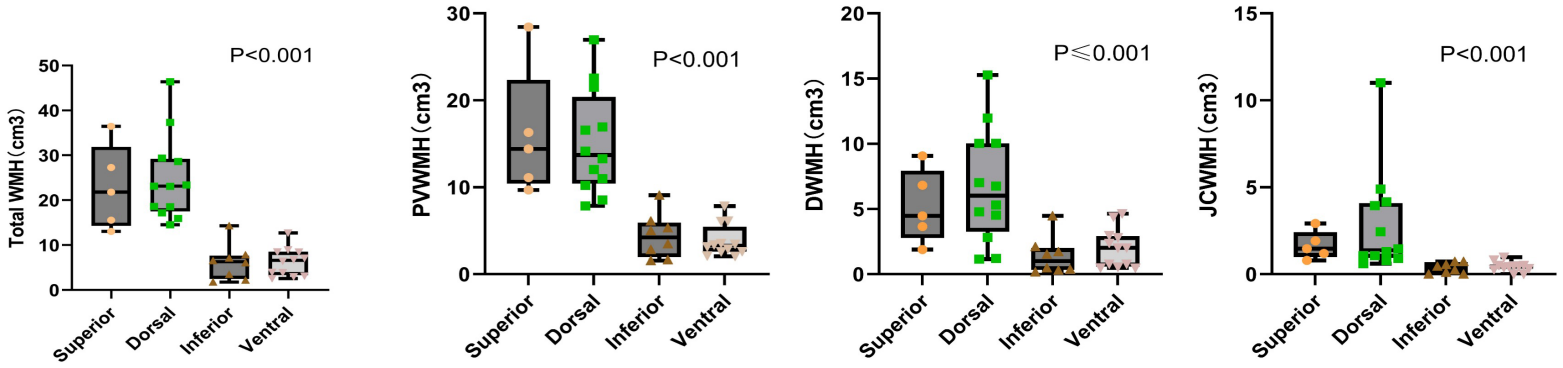
Comparison of WMH volume for different plaque distributions.

For patients with plaques, the difference between the four groups of total WMH volume, PVWMH volume, DWMH volume, and JCWMH volume was statistically significant. The total WMH volume, PVWMH volume, DWMH, and JCWMH volume were higher in patients with plaques on the superior and dorsal sides of the MCA M1 segment than in those with plaques on the inferior and ventral sides (Figure 5) (all *p* < 0.05).

### 3.4 Correlation of clinical and plaque characteristics with WMH volume

**Figure 6:**
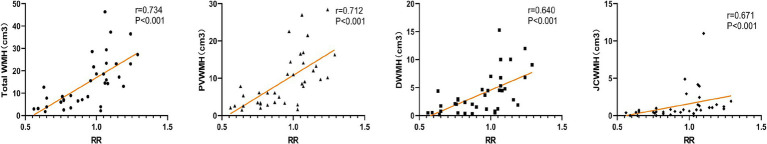
Correlation between RR and WMH volume.

For all patients, age, diabetes, and previous stroke events were positively correlated with total WMH volume, PVWMH volume, and JCWMH volume; for patients with plaques, plaque distribution, positive remodeling, eccentric plaques, and RR were positively correlated with total WMH volume, PVWMH volume, DWMH volume, and JCWMH volume (Table 2 and Figure 6) (all *p* > 0.05).

**Table 2:** Correlation of clinical and plaque characteristics with WMH volume.

Characteristic	Total WMH (cm^3^)		PVWMH (cm^3^)		DWMH (cm^3^)		JCWMH (cm^3^)	
	*r*	*p*-value	*r*	*p*-value	*r*	*p*-value	*r*	*p*-value
Clinical characteristics								
Age	0.388	<0.001	0.435	<0.001	0.204	0.051	0.353	0.001
Diabetes	0.274	0.008	0.277	0.008	0.162	0.123	0.263	0.011
Previous stroke events	0.344	0.001	0.322	0.002	0.284	0.006	0.432	<0.001
Diastolic blood pressure	−0.095	0.370	−0.104	0.322	−0.074	0.486	−0.218	0.037
Plaque characteristics								
Plaque	0.293	0.005	0.270	0.009	0.298	0.004	0.271	0.009
Plaque distribution	0.858	<0.001	0.853	<0.001	0.655	<0.001	0.818	<0.001
Moderate or severe stenosis	0.000	1.000	0.018	0.917	−0.150	0.374	0.115	0.498
T1 hyperintensity	−0.246	0.142	−0.201	0.232	−0.218	0.194	−0.112	0.509
Positive remodeling	0.710	<0.001	0.684	<0.001	0.642	<0.001	0.676	<0.001
Eccentric plaques	0.726	<0.001	0.705	<0.001	0.647	<0.001	0.676	<0.001
Plaque burden	0.197	0.242	0.199	0.238	0.108	0.524	0.335	0.042

### 3.5 Multifactor generalized linear model for WMH volume

Independent variables with *p* < 0.1 in Spearman’s correlation analysis were selected as control variables and included in the multifactor generalized linear model shown: the presence of plaques, plaque distribution, and positive remodeling were independent risk factors for the total volume of WMH (Table 3) (all *p* > 0.05).

**Table 3:** Multifactor generalized linear model for WMH volume.

Characteristic	Total WMH (cm^3^)		PVWMH (cm^3^)		DWMH (cm^3^)		JCWMH (cm^3^)	
	*B*	*p*-value	*B*	*p*-value	*B*	*p*-value	*B*	*p*-value
Plaque	4.614	0.008	2.415	0.026	1.634	0.011	0.632	0.019
Plaque distribution								
Superior or dorsal	9.446	0.004	6.683	0.002	1.751	0.253	0.995	0.284
Degree of stenosis								
Moderate or severe stenosis	1.631	0.482	0.972	0.518	0.259	0.812	0.383	0.558
Plaque vulnerability								
T1 hyperintensity	−5.219	0.220	−4.186	0.125	−0.738	0.709	−0.495	0.685
Positive remodeling	5.960	0.037	2.826	0.128	2.679	0.044	0.525	0.514
Eccentric plaques	2.785	0.301	1.189	0.496	1.274	0.302	0.203	0.789
Plaque burden	2.152	0.767	1.618	0.731	−0.564	0.865	1.507	0.459

## 4 Discussion

### 4.1 Findings of our study

Our study focused on specific characteristics of atherosclerotic plaques with less than 50% MCA stenosis, including plaque distribution, plaque burden, and remodeling patterns. Our findings confirm that, after adjusting for significant risk factors such as age and previous stroke events, the presence of plaques at the orifice of the perforating arteries of the MCA is significantly positively correlated with WMH volume. Additionally, among the 37 patients with arterial plaque formation, plaque distribution and positive remodeling are independent risk factors for the total volume of WMHs. Furthermore, patients with a higher vascular remodeling ratio tend to have a larger volume of WMHs.

### 4.2 Comparison with previous studies

The large artery is a new perspective to explore CSVD. A meta-analysis (3) involving 10,841 participants, which searched 21 eligible cross-sectional studies, found that the presence of both intracranial and extracranial atherosclerotic stenosis was associated with an increased risk of WMHs, but that this association was significant in the extracranial atherosclerotic stenosis (ECAS) but less so in the intracranial atherosclerotic stenosis (ICAS). Ni et al. (17), when assessing the relationship between WMHs burden and unilateral ICAS by examining luminal stenosis, plaque enhancement, and cerebral perfusion, concluded that the volume of DWMH ipsilateral to the site of ICAS was significantly greater than that of the contralateral site, which all suggests that ICAS contributes to the formation of WMHs. Another 3-year retrospective longitudinal study (18) yielded that patients with more severe ICAS were more likely to have progression of WMHs and had a significant correlation. Whereas the present study did not find an association between the degree of stenosis of the intracranial arteries and WMHs, probably because the MCA stenosis of our included subjects was <50% on MRA, which was not sufficient to cause a significant hypoperfusion of the cerebral blood flow, we did find that some of the plaque features were correlated with WMHs. Therefore, we speculate that plaque features are more valuable in assessing the progression of WMHs when intracranial arterial MRA shows stenosis <50%. These may provide clues to understand the mechanism of WMHs progression.

### 4.3 The presence of intracranial atherosclerotic plaques contributes to the progression of WMHs

The presence of intracranial atherosclerotic plaques in large arteries aggravated WMHs in our study. This finding is consistent with the results of Zhu and colleagues, who studied patients diagnosed with CSVD or embolic stroke of undetermined source (ESUS) (7) that intracranial non-stenotic atherosclerotic plaques, especially complex plaques, were strongly associated with CSVD imaging markers, while this study also found a significant association between intracranial atherosclerotic plaques (IAPs) and the presence of WMHs, which further suggests that non-stenotic plaques may contribute to white matter changes. The observed correlation could be due to the intracranial large arteries due to the lack of external elasticity plates, exhibit a markedly slower metabolism compared with the extracranial arteries, and a relatively slower autoregulatory function. As a result, the formation of cerebral aortic plaques often signals that a certain degree of injury already exists in small vessels throughout the body.

### 4.4 Plaques located superiorly or dorsally to the MCA M1 segment exhibit a greater burden of WMHs

Our study enrolled patients with less than 50% stenosis of the MCA, who are traditionally classified as having small vessel occlusions. These penetrating arteries, which include the lenticulostriate arteries (LSA), usually originate at acute angles from the parent artery. This anatomical feature predisposes to the formation of atheromatous plaque. This region is also one of the common sites for leukoaraiosis (LA) lesions, considered one of the manifestations of CSVD. A previous study focusing on basilar artery branching atherosclerotic disease (BABAD) found (19) that patients with BABAD had a higher severity of WMHs in the periventricular region of the brain, and also demonstrated a significant association between WMHs and BABAD, and this association was independent of vascular risk factors. Our study reached similar conclusions, showing that atherosclerosis in penetrating arteries is significantly correlated with WMH volume. The recent ZOOM@SVDs study utilized the advantage of 7T MRI to show the fine structural function of small perforating vessels and found that perforating arteries were strongly associated with CSVD (20). Another cohort study demonstrated that ischemic stroke patients with BAD are often accompanied by a high prevalence of cerebral white matter lesions (21).

Our results showed that total WMH volume and PVWMH volume were greater in patients with plaques located on the superior or dorsal side of the MCA. After also controlling for the plaque intrinsic characteristics variable, plaques located on the superior or dorsal side remained an independent risk factor for total WMH volume, suggesting that it is plaque distribution rather than plaque intrinsic characteristics that determine the progression of WMHs. Our study contributes to further understanding of the pathophysiology of WMHs and thus monitors the relationship between cerebral atherosclerosis and WMHs. However, the relatively small sample size of this study may have led to insufficient statistical power and an increased risk of Type II errors, so caution is needed in interpreting these results. Therefore, we used HR-MRI to detect atherosclerotic plaques in patients’ MCA branch arteries and concluded that patients with plaques located superiorly or dorsally to the MCA M1 segment had a greater WMHs burden, and the correlation may be because the perforating arteries often open superiorly or dorsally to the MCA (22), and plaque located at the orifice of a perforating artery often leads to altered hemodynamics (23) in the downstream region, which in turn causes ischemic damage to white matter. Not only can this ischemia lead to oligodendrocyte death and axonal degeneration, causing an increase in white matter volume, but reduced cerebral perfusion can lead to impaired cerebrovascular reactivity (24), which is the ability of the cerebral vessels to dilate in response to changes in metabolic demand, can lead to inefficient cerebral blood flow regulation. This impairment can contribute to white matter ischemia and atrophy. This is not a simple CSVD, but a result of atherosclerosis of the large arteries.

### 4.5 Positive remodeling increases the WMHs burden

Previous studies have found that structural cranial arterial remodeling may be important in the pathophysiology of WMHs (25). Chen et al. concluded that cerebral arterial remodeling scores are positively associated with severe WMHs and that patients with large cranial arterial diameters may have a heavier CSVD burden (26). It has also been suggested that cerebral arterial outward remodeling may be a biomarker of systemic arterial stiffness (27). A large number of recent research has established a close association between cerebral arterial stiffness and the development of CSVD (28), which indirectly suggests that cerebral arterial remodeling about WMHs. Meanwhile, a recent systematic evaluation and meta-analysis showed that intracranial arterial dilatation was associated with a high prevalence of CSVD imaging markers in patients with ischemic stroke (29). Our study further established a close correlation between plaque vulnerability parameters (i.e., positive remodeling) and WMHs. The correlation may be because intracranial large arteries, as a bridge between extracranial large arteries and small cerebral vessels, function to reduce systemic pressure and pulsatility of blood delivery to cerebral capillaries, and cerebral arteriolar ectopic remodeling may reduce physiological cerebral self-regulation, leading to the transmission of hemodynamic loads to the downstream small blood vessels, thus causing CSVD (30). Thus, our findings suggest that remodeling of cranial arteries may play an important role in the development of large artery disease and small vessel disease.

Outward remodeling of intracranial arteries, as an early response to atherosclerotic plaque formation, has the potential to delay luminal stenosis but also increases the risk of plaque rupture and has unknown effects on the brain. Previously Zhang et al. (31) in their study of the relationship between remodeling patterns and ischemic stroke in patients with atherosclerotic MCA stenosis, found that positive remodeling was associated with an increase in plaque area, whereas this suggests that the atherosclerotic disease burden of the MCA is increasing, and that an increase in the plaque burden further increases the overall cerebrovascular event risk. Whereas the correlation between atherosclerotic changes in intracranial arteries without significant stenosis on MRA and small vessel disease has rarely been explored, our study focused on examining the remodeling of intracranial arterial walls, looking in particular for evidence of positive remodeling (i.e., outward expansion of the arterial wall) and discussing in-depth the impact of the pattern of remodeling on the progression of white matter, and the results of the present study suggest that positive remodeling may be associated with more severe WMHs.

### 4.6 Potential limitations

The present study has some limitations: (1) the sample size of this study was limited, and although multifactor analysis revealed a close association between plaque characteristics and WMHs, a small number of data points are more susceptible to random fluctuations. This affects the robustness of the statistical analysis and restricts the generalizability of the findings. Future studies should aim to expand the sample size to better assess the true relationship between atherosclerotic plaques and WMHs; (2) the retrospective design of the present study does not allow for the assessment of the causal relationship between intracranial atherosclerosis and WMHs, whereas prospective and longitudinal follow-up studies help to observe the dynamic course of the relationship between plaque characteristics and WMHs; (3) the present study lacks hemodynamic indices, and future studies may investigate whether plaque distribution, positive remodeling, and other plaque characteristics affecting white matter lesions are mediated by hemodynamic factors; (4) the present study did not explore CSVD imaging markers other than WMHs, limiting the comprehensive assessment of cranial atherosclerosis’s impact on brain injury.

## 5 Conclusion

In conclusion, our study found that the presence of atheromatous plaques, their geometric distribution, and positive remodeling were closely associated with the volume of WMHs, contributing to the progression of white matter lesions. HR-MRI provided *in vivo* evidence that intracranial atherosclerosis, particularly plaque characteristics, can lead to white matter lesions, which further demonstrated that atherosclerotic alterations in the brain may contribute to the development of WMHs. Therefore, more attention should be paid to the interconnectedness of cerebral large-vessel and CSVD. HR-MRI can also serve as a valuable tool for assessing plaque characteristics, aiding in the stratification of future clinical risk and supporting the development of individualized treatment strategies.

## Data Availability

The raw data supporting the conclusions of this article will be made available by the authors, without undue reservation.
